# Characterization of Five ECF Sigma Factors in the Genome of *Pseudomonas syringae* pv. syringae B728a

**DOI:** 10.1371/journal.pone.0058846

**Published:** 2013-03-14

**Authors:** Poulami Basu Thakur, Vanessa L. Vaughn-Diaz, Jessica W. Greenwald, Dennis C. Gross

**Affiliations:** Department of Plant Pathology and Microbiology, Texas A&M University, College Station, Texas, United States of America; University of the West of England, United Kingdom

## Abstract

*Pseudomonas syringae* pv. syringae B728a, a bacterial pathogen of bean, utilizes large surface populations and extracellular signaling to initiate a fundamental change from an epiphytic to a pathogenic lifestyle. Extracytoplasmic function (ECF) sigma (σ) factors serve as important regulatory factors in responding to various environmental signals. Bioinformatic analysis of the B728a genome revealed 10 ECF sigma factors. This study analyzed deletion mutants of five previously uncharacterized ECF sigma factor genes in B728a, including three FecI-type ECF sigma factors (ECF5, ECF6, and ECF7) and two ECF sigma factors placed in groups ECF11 and ECF18. Transcriptional profiling by qRT-PCR analysis of ECF sigma factor mutants was used to measure expression of their associated anti-sigma and outer membrane receptor proteins, and expression of genes associated with production of extracellular polysaccharides, fimbriae, glycine betaine and syringomycin. Notably, the B728aΔ*ecf7* mutant displayed reduced swarming and had decreased expression of CupC fimbrial genes. Growth and pathogenicity assays, using a susceptible bean host, revealed that none of the tested sigma factor genes are required for *in planta* growth and lesion formation.

## Introduction

*P. syringae* pv. syringae B728a is a highly versatile foliar pathogen of bean that causes brown spot, a disease manifested as water-soaked lesions on bean leaves and pods [1]. The Gram-negative bacterium can effectively survive as an epiphyte on bean leaf surfaces prior to aggressively invading the apoplastic tissues [1]. Strain B728a grows to substantial numbers on leaf surfaces before entering the host through wound sites or natural openings, such as stomata. Thus, in order to adapt to the diverse environments encountered during epiphytic growth and plant pathogenesis, the bacterium has a critical need to sense and quickly respond to its extracellular environment. Extracytoplasmic function (ECF) sigma factors, functioning as transcriptional regulators of gene expression in response to specific environmental signals, offer a convenient regulatory mechanism for the rapid activation of genes in response to fluctuating environmental conditions [2], [3].

**Figure 1:**
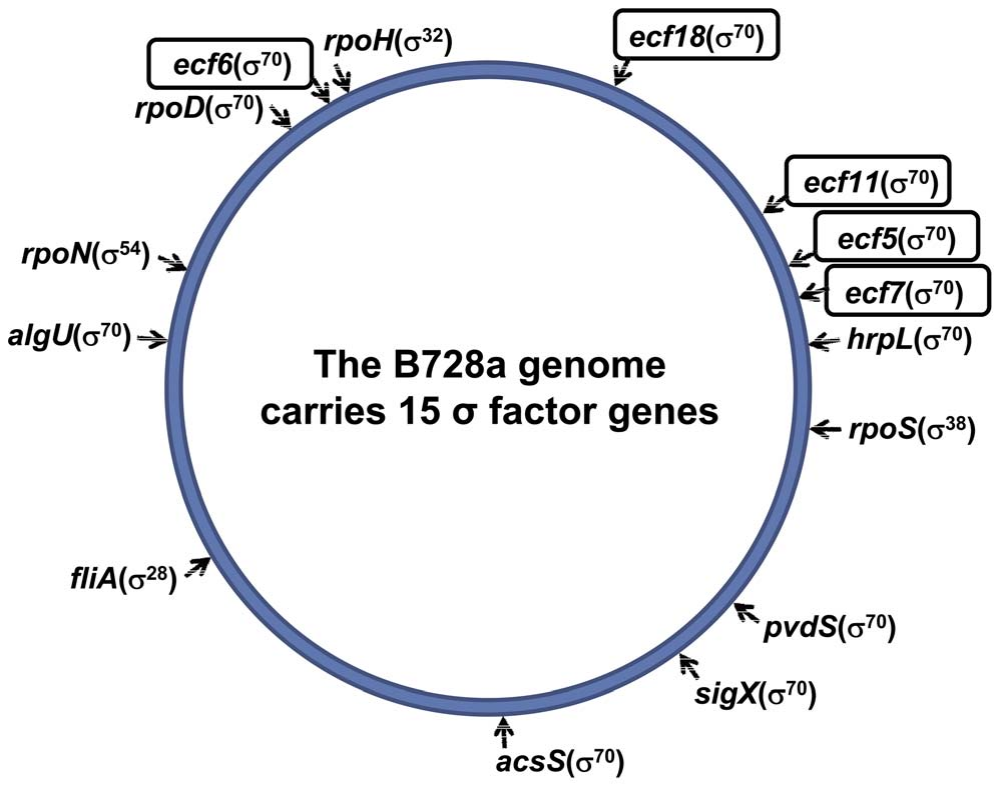
Schematic representation of the B728a genome showing the different sigma factors. The 6.09 Mb genome of B728a encodes 15 distinct sigma factors. *rpoD* (Psyr_4641) is the essential housekeeping sigma factor for this bacterium belonging to the σ^70^ family, while *rpoN* (Psyr_4147) belongs to the σ^54^ type of sigma factors. The other sigma factors belong to the alternative sigma factor family and include *fliA* (Psyr_3437), which controls the flagellar biosynthesis genes; *rpoS* (Psyr_1374), the starvation phase σ factor; *rpoH* (Psyr_4748), the heat shock sigma factor; and 10 ECF sigma factors, which are summarized in Table 2. Five of these belong to the FecI-type of ECF sigma factors, including *ecf5* (Psyr_1040), *ecf7* (Psyr_1107), *pvdS* (Psyr_1943), *acsS* (Psyr_2580), and *ecf6* (Psyr_4731). The five ECF sigma factors characterized in this study are identified by rectangular boxes.

The sigma factor class of proteins initiate gene transcription in bacteria by reversibly binding to the multi-subunit core of RNA polymerase [4]. By binding to the polymerase, sigma factors provide specificity to promoter recognition and contribute to DNA strand separation [5]. Sigma factors can be grouped into two major categories, the σ^70^ and the σ^54^ type proteins. The domain architecture of the σ^70^ family and σ^54^ relative to transcriptional initiation is reviewed by Österberg et al. [6] and Gruber and Gross [5]. Although members of the σ^54^ family are widespread among bacteria, most bacterial genomes encode multiple proteins that are homologous to σ^70^ and a single representative homologous to σ^54^ (called RpoN) [3], [5]. All *Pseudomonas* species have one housekeeping sigma factor, RpoD (σ^70^), that controls the basal expression level of most genes during exponential growth and a variable number of alternative sigma factors that possess different promoter-recognition properties [5], [7]. The genome of *P. syringae* pv. syringae B728a (Fig. 1) carries a total of 15 sigma factor genes that in addition to *rpoD* and *rpoN*, includes *fliA* (σ^28^) associated with flagellin biosynthesis [8], *rpoS* (σ^38^) associated with stationary phase growth and autoinduction [9], *rpoH* (σ^32^) associated with heat-shock induction [3], and 10 ECF (σ^70^) sigma factors [10]. Bacterial cells can alter their response to environmental stimuli by the activation of a specialized ECF sigma factor to change its transcriptional course [5]. When cells are not exposed to the stimulus, most alternative sigma factors remain inactive by directly interacting with a specific anti-sigma factor protein [7].

It is generally observed that bacterial genomes harboring a greater number of ECF sigma factors are associated with bacteria from distinctive environments with complex lifestyles [10]. Sequenced genomes of fluorescent pseudomonads reveal the frequent occurrence of ECF sigma factors [10]–[12]. For example, the genomes of *P. aeruginosa* PAO1 and *P. putida* KT2440 are similar in size and each encodes 19 ECF sigma factors [13], whereas the genome of *P. fluorescens* Pf-5 encodes 27 ECF sigma factors [14]. In contrast, 10 ECF sigma factors have been identified in the completely sequenced genomes of three *P. syringae* pathovars [10], including *P. syringae* pv. syringae B728a (Fig. 1). Of these ECF sigma factors, five, including HrpL, AlgU, and SigX, are stress response sigma factors, whereas the other five (i.e., PvdS, AcsS, ECF5, ECF6, and ECF7) are members of the FecI-like iron responsive group of sigma factors [15]. A distinctive feature of the iron responsive ECF sigma factors is the genomic arrangement of a FecR-like transmembrane sensor gene and a specific FecA-like outer membrane protein gene located near the respective ECF sigma factor gene [10], [16]. The existence of multiple FecI-type ECF sigma factors in *Pseudomonas* spp. is indicative of their role in regulating different iron transport systems [3], [10], [16].

A limited number of ECF sigma factors, including HrpL, AlgU, SigX, PvdS, and AcsS, have been characterized in *Pseudomonas* [3], [10], [17]; the genome of *P. syringae* B728a encodes these five ECF sigma factor genes and five other ECF genes that remain uncharacterized [10]. HrpL activates the expression of the well-known *hrp/hrc* encoded type III secretion system and effector proteins that have a major role in plant pathogenicity of *P. syringae* [18], [19]. Characteristically, *hrpL* mutants show a conspicuous phenotype whereby they fail to cause disease symptoms in host plants. The AlgU stress response ECF sigma factor, also called AlgT [20], is required for alginate production in *P. syringae* and increases tolerance to toxic compounds, such as copper, in the leaf environment [20]–[22]. The SigX ECF sigma factor regulates expression of a gene encoding a major outer membrane protein, OprF, in *P. aeruginosa* and the root-colonizing *P. fluorescens* [23]. The OprF protein is a nonspecific porin involved in maintaining cell shape and growth in low-osmolarity environments [24]. Another familiar ECF sigma factor, PvdS, is associated in fluorescent pseudomonads with the regulation of genes involved in the biosynthesis of the siderophore, pyoverdine [2], [25], [26]. Recently, Greenwald et al. [17] described AcsS, an ECF sigma factor associated with controlling the biosynthesis and secretion of achromobactin, a second siderophore produced by *P. syringae* strain B728a.

The goal of this study, was to generate deletion mutants for each of the five uncharacterized ECF sigma factor genes of *P. syringae* pv. syringae B728a and characterize the ECF mutant phenotypes, especially in relation to survival as a plant-associated bacterium. Based on an investigation by Staroń et al. [27], who classified ECF sigma factors into over 40 groups based on their modular architecture, the ECF sigma factors of strain B728a were identified by ECF group and named according to the report’s guidelines. Because iron availability exerts a strong influence on the expression of several virulence associated factors in *P. syringae* pv. syringae B728a [15], [28], we characterized the roles played by three members of the FecI-type of ECF sigma factors in shaping the B728a host-pathogen interaction. We demonstrate that expression of the FecI-like ECF sigma factors is significantly up-regulated in conditions of iron stress and down-regulated in high iron conditions. In addition, ample quantities of iron are required for the production by B728a of a cyclic lipopeptide phytotoxin, syringomycin, which contributes to virulence [29]. Because earlier studies of sigma factor mutants (i.e. *rpoS*, *acsS*, *pvdS*, and *hrpL*) in *P. syringae* pv. syringae did not reveal a regulatory effect on expression of the phytotoxin genes [17], [30], a significant goal was to resolve whether the remaining uncharacterized ECF sigma factors included syringomycin production as a regulatory target. In addition to the three FecI-like ECF sigma factor genes, two ECF sigma factors placed in groups ECF11 and ECF18 [27] were characterized for their contributions to pathogen resistance to specific environmental stresses. Transcriptional profiling by qRT-PCR analysis of ECF sigma factor mutants measured the expression of genes encoding their associated anti-sigma and outer membrane receptor proteins, as well as the expression of genes associated, for example, with production of extracellular polysaccharides, fimbriae, and glycine betaine. Ultimately, each ECF sigma factor mutant was evaluated for growth and pathogenicity in leaves of a susceptible bean host.

## Materials and Methods

### Bacterial strains and media

The strains of *P. syringae* pv. syringae and *E. coli* used in this study are recorded in Table 1. The *E. coli* Mach1 T1 cells were used following topoisomerase reactions as described by the manufacturer (Invitrogen, Carlsbad, Calif., USA). Routine culturing of *P. syringae* pv. syringae strains occurred at 25°C in nutrient broth-yeast extract (NBY) liquid or agar medium [31], or on King’s B (KB) agar medium [32]. Assays for syringomycin production were conducted on Hrp minimal medium (HMM) agar [17], [33]. Mannitol glutamate-yeast extract (MGY) agar supplemented with 0.6 M sorbitol or MGY supplemented with 5% sucrose was used in assays for mucoidy [20]. Swarming activity was measured on NBY medium containing 0.4% agar [34]. The following antibiotic concentrations ( µg/ml) were used: rifampicin, 100; kanamycin, 75; tetracycline, 20; ampicillin, 100; gentamycin, 5; and spectinomycin, 100.

**Table 1:** Strains and plasmids used in this study.

Designation	Relevant Characteristics	Source
***E. coli***		
DB3.1	F^-^ *gyrA462 endA1 glnV44* Δ(*sr1-recA*) *mcrB mrr hsdS20*(rB^-^ mB^-^) *ara14 galK2 lacY1 proA rpsL20*(Sm^r^) *xyl5* Δ*leu mtl1*	[69]
Mach1 T1	F^-^ Δ*recA1398 endA1 tonA* φ80(*lacZ*)ΔM15 Δ*lacX74 hsdR*(rK^-^ mK^+^)	Invitrogen
SW105	DY380 (*cro*-*bioA*) <>*araC*-P_BAD_ *Cre* Δ*galK*	National Cancer Institute
***P. syringae*** **pv. syringae**		
B728a	Wild-type, bean pathogen; Rif^r^	[70]
B728aΔ*ecf5*	*ecf5* mutant derivative of B728a, Rif^r^	This study
B728aΔ*ecf7*	*ecf7* mutant derivative of B728a, Rif^r^	This study
B728aΔ*ecf18*	*ecf18* mutant derivative of B728a, Rif^r^	This study
B728aΔ*ecf11*	*ecf11* mutant derivative of B728a, Rif^r^	This study
B728aΔ*ecf6*	*ecf6* mutant derivative of B728a, Rif^r^	This study
B728aΔ*gacS*	*gacS* mutant derivative of B728a, Rif^r^	[38]
BR132	*syrB1*::Tn*3*HoHo1 derivative of B301D-R; Pip^r^ Rif^r^	[42]
**Plasmids**		
pBH474	*flp* constitutively expressed; Gm^r^ Suc^s^	[71]
pENTR/D-TOPO	Gateway entry vector; Km^r^	Invitrogen
pE0362	pENTR/D-TOPO carrying *ecf18*, Km^r^	This study
pE0892	pENTR/D-TOPO carrying *ecf11*, Km^r^	This study
pE1040	pENTR/D-TOPO carrying *ecf5*, Km^r^	This study
pE1107	pENTR/D-TOPO carrying *ecf7*, Km^r^	This study
pE4731	pENTR/D-TOPO carrying *ecf6*, Km^r^	This study
pKD13	Template plasmid with FRT-flanked *nptII*	[72]
pLVCD	Gateway destination vector for mating with *P. syringae*; pBR322 derivative with *mob* genes from RSF1010; Tc^r^ Ap^r^ Cm^r^	[41]
pLV0362	pLVCD carrying *ecf18*; Tc^r^ Ap^r^	This study
pLV0892	pLVCD carrying *ecf11*; Tc^r^ Ap^r^	This study
pLV1040	pLVCD carrying *ecf5*; Tc^r^ Ap^r^	This study
pLV1107	pLVCD carrying *ecf7*; Tc^r^ Ap^r^	This study
pLV4731	pLVCD carrying *ecf6*; Tc^r^ Ap^r^	This study
pLV0362-FP	pLVCD carrying upstream and downstream regions of *ecf18* fused to *nptII*; Tc^r^ Ap^r^ Km^r^	This study
pLV0892-FP	pLVCD carrying upstream and downstream regions of *ecf11* fused to *nptII*; Tc^r^ Ap^r^	This study
pLV1040-FP	pLVCD carrying upstream and downstream regions of *ecf5* fused to *nptII*; Tc^r^ Ap^r^ Km^r^	This study
pLV1107-FP	pLVCD carrying upstream and downstream regions of *ecf7* fused to *nptII*; Tc^r^ Ap^r^ Km^r^	This study
pLV4731-FP	pLVCD carrying upstream and downstream regions of *ecf6* fused to *nptII*; Tc^r^ Ap^r^ Km^r^	This study
pPROBE-KT’	Promoter-probe vector with pVS1/p15a replicon and *gfp* reporter, Km^r^	[73]
pPKT::*ecf7*	pPROBE-KT’ carrying *ecf7* along with 114 bp upstream; Km^r^	This study
pRK2073	Helper plasmid; Sp^r^ Trm^r^	[37]

### General DNA manipulations

New England Biolabs (Beverly, Mass., USA) was the source of restriction enzymes, T4 DNA ligase, and Phusion high-fidelity DNA polymerase. Oligonucleotides were designed using the PrimerQuest and OligoAnalyzer applications of Integrated DNA Technologies (Coralville, Iowa, USA) and purchased from them. The primer sequences used in this study are listed in Table S1. Once target genes were amplified by PCR they were cloned into the pENTR/D-TOPO vector (Invitrogen) using the Gateway technology [35]. Recombination between pENTR constructs and Gateway destination vectors was mediated by LR clonase (Invitrogen) according to the manufacturer’s instructions. Chemical transformation or electroporation was used to introduce plasmids into *E. coli* [36]. Tri-parental mating with the helper plasmid pRK2073 [37] was used to introduce plasmids into *P. syringae* pv. syringae. PCR procedures used standard cycling conditions.

### Construction of markerless *ecf* gene deletions in B728a

Precise deletion mutants of five ECF σ factor genes, *ecf5* (Psyr_1040), *ecf7* (Psyr_1107), *ecf6* (Psyr_4731), *ecf11* (Psyr_0892), and *ecf18* (Psyr_0362), were created in strain B728a using a modified [17], [38] Red recombinase deletion method [39]. This procedure utilizes homologous recombination in *E. coli* SW105 (http://recombineering.ncifcrf.gov), which is mediated by recombination proteins provided from a defective λ prophage inserted into the SW105 genome [40]. Recombination proteins are transcribed from a promoter, which is repressed by the temperature-sensitive repressor *cI*857 at 32°C and de-repressed at 42°C. No recombination proteins are produced when bacteria containing the defective λ prophage are kept at 32°C, but are produced after a brief (15–20 min) heat-shock at 42°C.

In this strategy, a genomic fragment containing the *ecf* gene-of-interest (GOI) along with 3–4 kb of flanking DNA was PCR amplified using a Phusion*®* long range proof-reading polymerase (ThermoScientific F-553S). The PCR primers used to amplify the five *ecf* genes are listed in Table S1. The amplified PCR product was then directionally cloned into a TOPO cloning vector, pENTR/D-TOPO (Invitrogen) according to the manufacturer’s instructions. LR clonase II (Invitrogen) was used to carry out recombination between the pENTR construct and the Gateway destination vector, pLVC-D [41]. The pLVC-D plasmid was then introduced into SW105 cells via electroporation. A linear kanamycin cassette (FRT-Km-FRT) flanked on either side by FLP recognition target (FRT) sites, was amplified from the pKD13 plasmid using PCR primers with 36 bp extensions that were homologous to regions adjacent to the GOI. This linear cassette was moved into SW105 by electrotransformation following heat shock at 42°C for 15 minutes. Homologous recombination mediated by the phage recombination proteins produced as a result of the brief heat shock replaced the GOI with the FRT-Km-FRT cassette. The pLVC-D plasmid with the linear cassette was then introduced into B728a via triparental mating using the helper plasmid pRK2073 [37]. The kanamycin marker was later removed from mutant B728a by introducing the pBH474 vector carrying FLP recombinase.

### Syringomycin assays

The production of syringomycin by B728a and derivative strains was evaluated on HMM agar [38]. Bacteria were grown overnight in 2 ml NBY at 25°C with shaking. Cells were washed and resuspended in sterile water to OD_600_ = 0.3 (∼2×10^8^ CFU/ml), and spotted on HMM agar. The strains were incubated at 25°C for 3 days, following which they were finely sprayed with a suspension of *Geotrichum candidum* strain F-260 using sterile chromatography sprayers [42]. Inhibition zones were measured a day later. Previously, it was demonstrated that syringomycin is the only metabolite produced by *P. syringae* pv. syringae inhibitory to *G. candidum* [42]. Consequently, mutant strain BR132 [43], which is specifically mutated in the *syrB1* synthetase gene for syringomycin, was used along with B728aΔ*gacS* [38] as negative controls for antifungal activity. The experiment was repeated three times.

### Swarming motility assays

Swarming motility of the five *ecf* mutant strains was compared to parental strain B728a and its non-swarming derivative, B728aΔ*gacS*, after culturing on semisolid NBY containing 0.4% agar [20]. Strains were initially grown overnight at 25°C with shaking in 2 ml of NBY. Cells were pelleted, washed once with fresh NBY, and resuspended in NBY. Fresh NBY (5 ml) was then seeded with 6 µl of the washed culture and grown at 25°C with shaking, up to OD_600_ = 0.3. Sterile filter discs (Fisherbrand, Grade P8-Creped) sized to 6 mm with a standard 1-hole punch, were placed in the center of each plate [38]. The discs were inoculated with ∼2×10^8^ CFU/ml and the plates then were incubated at 25°C for 24 h in a moist chamber. Swarming distances were measured from the outer edge of the filter paper. Each strain was tested on three semisolid NBY agar plates, and the experiment was repeated three times.

### Shock assay to assess the tolerance of the *ecf18* mutant to toluene

Tolerance to toluene was determined for B728aΔ*ecf18* as compared to B728a in NBY liquid medium using the procedure described by Duque et al. [44]. The bacterial strains were cultured overnight in of NBY (5 ml) with shaking at 26°C. From the overnight cultures, 500 µl were used to inoculate 20 ml of fresh NBY liquid medium, and incubation conditions were continued until a final cell concentration of 2×10^8^ CFU/ml. Cultures were divided into two 10-ml portions. Toluene (Sigma) was added (30 µl) to one culture to achieve a final concentration of 0.3% (vol/vol) and the other culture received sterile water (30 µl) as a control. Optical density measurements (OD_600_ nm) and viable cell counts on NBY agar were determined before toluene was added, and subsequently at 10, 30, 60, 120, and 180 min after exposure to toluene. These assay results represent the average of three biological replicates.

### Assay of *ecf11* mutant for oxidative stress resistance

Resistance to oxidative stress was measured by the disk diffusion inhibition assay as described by Matsumoto et al. [45]. The level of resistance to hydrogen peroxide was tested for strains B728aΔ*ecf11* and B728a spread on HMM agar plates by exposing them to H_2_O_2_ applied to a paper disk (10 µl of 100 mM H_2_O_2_). After incubation for 48 h at 25°C °C, the zones of growth inhibition were measured; each experiment was independently repeated twice with three biological replicates.

### Pathogenicity assays in bean leaves

The ability of the five B728a ECF sigma factor mutants (B728aΔ*ecf5*, B728aΔ*ecf6*, B728aΔ*ecf7*, B728aΔ*ecf11*, and B728aΔ*ecf18*) to cause disease on bean plants was evaluated by methods described previously [17], [38]. B728a and derivative sigma factor mutants were grown at 25°C with shaking overnight in 2 ml of NBY. The overnight cultures were used to inoculate 100 ml of fresh NBY medium, and then further incubated at 25°C to an OD_600_ = 0.6. Cultures were pelleted, washed with sterile deionized water, and resuspended in sterile deionized water to a final concentration of ∼5×10^6^ CFU/ml. Two-week-old Blue Lake 274 bean plants (*Phaseolus vulgaris* L.) were vacuum infiltrated with a bacterial suspension or sterile deionized water as described previously [17], [38]. The excess inoculum was gently washed off plants with sterile water, leaves were allowed to air dry, and then plants were maintained at 22°C under fluorescent plant growth lights. Disease symptoms were evaluated 2–3 days post inoculation relative to parental strain B728a. Each ECF sigma factor mutant, along with strains B728a and B728aΔ*gacS* as controls, was tested on five individual bean plants; the experiment was independently repeated twice.

To evaluate the growth of ECF sigma factor mutants in bean leaves, bacterial populations were measured at 0, 2, 4, and 6 days after vacuum infiltration. Populations of the mutants were compared to the fully virulent parental strain B728a and the nonpathogenic derivative B728aΔ*gacS* mutant. After inoculation by methods described above, five leaves were arbitrarily collected from each plant. A 2-ml screw-cap microcentrifuge tube (BioPlas Inc., San Rafael, Calif., USA) was used to punch out 14 leaf discs per leaf (8 mm diameter). The discs were homogenized by mortar and pestle in a Silwet phosphate magnesium buffer [17]. Serial dilutions were made in sterile water, and spread on KB agar medium. Populations were calculated after enumeration of colonies on plates incubated at 25°C for 48 h.

### RNA isolation for qRT-PCR studies

Methods for analysis of the influence of *ecf* sigma factor deletions on gene expression by quantitative real-time reverse-transcription PCR (qRT-PCR) are as described by Greenwald et al. [17]. B728a and derivative mutant strains were grown to late log phase OD_600_ = 0.6 (∼5×10^8^CFU/ml) at 25°C in NBY shake cultures. For studies on the influence of iron on gene expression, overnight cultures of strains were pelleted and washed in iron-free water, and then grown (OD_600_ = 0.6, 25°C) in modified liquid HMM without added iron, or HMM medium supplemented with either 10 or 100 µM FeCl_3_. Iron-free glassware was used to culture bacteria to minimize iron contamination. A total of three technical replicates of three biological samples were prepared for each condition. RNAprotect reagent (Qiagen Inc., Valencia, Calif., USA) was used to fix cultures, and the total RNA was extracted using the RNeasy*®* Mini Kit as per the manufacturer’s instructions (Qiagen). RNA of high quality was measured (RIN above 8.0) [17] using an Agilent 2100 Bioanalyzer (Agilent Technologies, Inc.) and selected; total RNA samples were quantified using micro-spectrophotometry (Nano-Drop Technologies, Inc.). RNA samples were treated with TURBO™ DNAse (Ambion, Austin, Texas, USA) to remove any residual DNA in the samples. cDNA was generated using the SuperScript*®* VILO™ synthesis kit (Invitrogen) as described by Greenwald et al. [17], and diluted to 10 ng/ µl. Reverse transcription was conducted by incubating samples at 25°C for 10 minutes, followed by an incubation at 42°C for 60 minutes. The reaction was terminated by incubating the samples at 85°C for 5 minutes.

### qRT-PCR analyses

**Figure 2:**
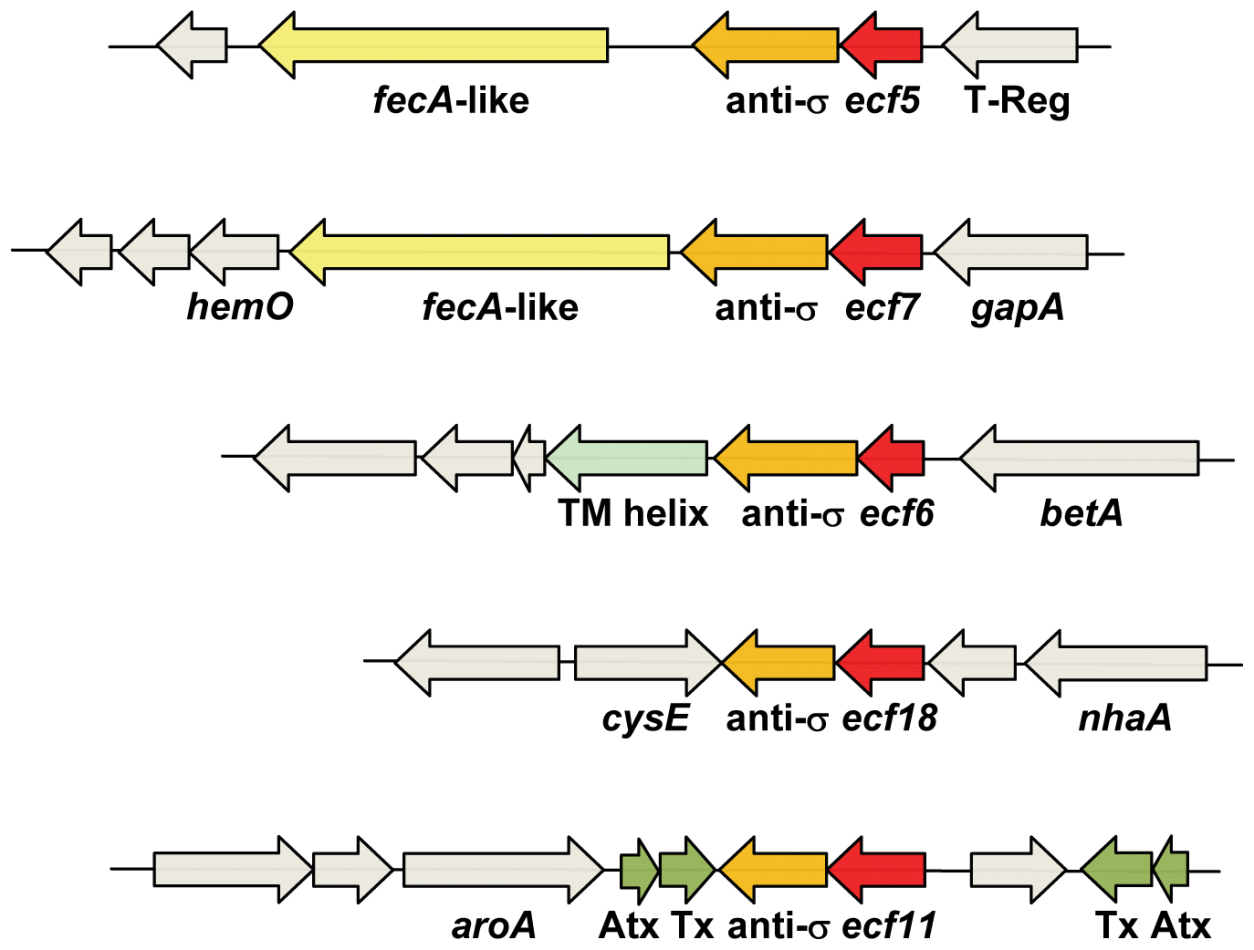
Genomic neighborhood of *ecf5*, *ecf7*, *ecf6*, *ecf18*, and *ecf11* sigma factor genes. The genes for ECF sigma factor genes are shown in red, the anti-sigma factor genes in brown, the FecA-like genes in yellow, a transmembrane (TM) helix protein in light green, and the toxin/antitoxin (Tx/Atx) genes in olive green; other nearby genes are shown in gray. Additional genes are labeled as T-Reg, transcriptional regulator; *gapA*, glyceraldehyde-3-phosphate dehydrogenase; *betA*, choline dehydrogenase; *nhaA*, Na+/H+antiporter NhaA; *cysE*, serine O-acetyltransferase; and *aroA*, 5-enolpyruvylshikimate-3-phosphate synthase.

An Applied Biosystems 7500 Fast Real-Time PCR System was used in conjunction with the SYBR*®* Green ER™ Reagent System of Invitrogen for qRT-PCR analyses. Each 20 µl reaction mixture contained 10 µl of the SYBR*®* Green ER™ SuperMix Universal, 8.16 µl nuclease-free water, 0.4 µl of both the forward and reverse primers (200 nM final), 1 µlof template DNA (10 ng/ µl), and 0.04 µl ROX reference dye. All qRT-PCR primers are listed in Table S2. The linearity of detection was confirmed to have a correlation coefficient of at least 0.98 (r^2^>0.98) for each primer pair by measuring a 5-fold dilution curve with cDNA generated from reverse transcription of bacterial RNA. Conditions for qRT-PCR consisted of incubation at 95°C for 20 seconds, followed by 40 cycles involving 3 seconds at 95°C and 30 seconds at 60°C. This was confirmed by melting curve analysis. Gene expression was normalized to that of the housekeeping genes, *recA* and *16s-rRNA* [17], and all primer pairs amplified a single primer product. qRT-PCR was performed to determine the effects of low and high iron conditions on the expression of three FecI-like sigma factor genes *ecf5* (Psyr_1040), *ecf7* (Psyr_1107), and *ecf6* (Psyr_4731) in parental strain B728a as compared to *ecf* gene deletion mutants. The effects of deletion of either the *ecf5*, *ecf6* or *ecf7* genes on expression of their associated putative transmembrane sensor or outer membrane receptor genes (Fig. 2) were also analyzed using primers (Table S2) specific to Psyr_1039, Psyr_1038, Psyr_1105, Psyr_1106, Psyr_4730, and Psyr_4729. Additional primers (Table S2) were developed for a potential heme oxygenase gene (Psyr_1104) associated with *ecf7*, and for the betaine genes *betA* (Psyr_4732) and *betB* (Psyr_4733) associated with *ecf6*. To determine the effects of the *ecf7* gene deletion on downstream fimbrial-type genes, primers specific to Psyr_1131-Psyr_1134 were used to measure transcript abundance.

A comparative C_t_ (cycle threshold) method [7], also known as the ΔΔC_t_ method, was used to determine an increase or decrease in transcript levels. Fold change in gene expression was calculated according to the 2^-ΔΔCt^ equation, where 2^-ΔΔCt^ = [(C_t__gene-of-interest_ – C_t__internal control_) Treated sample – (C_t__gene-of-interest_ – C_t__internal control_) Untreated sample] [7]. A 2-fold or more change in C_t_ for the sample of interest as compared to the control sample was considered to be significant [38]. The actual decrease in fold change was computed by taking the negative inverse of the fold change value [7].

## Results

### Organization and classification of five B728a genes encoding ECF sigma factors

An investigation of the predicted protein products of Psyr_0362, Psyr_0892, Psyr_1040, Psyr_1107, and Psyr_4731 using a combination of the bioinformatic resources Pfam (http://pfam.sanger.ac.uk/) and InterProScan (http://www.ebi.ac.uk/Tools/pfa/iprscan/) revealed the absence of the σ_3_ and the presence of the σ_2_ and σ_4_ conserved domains, which are signature characteristics of ECF sigma factors [6], [27]. A conserved domain search using NCBI Conserved Domains database (http://www.ncbi.nlm.nih.gov/cdd) also confirmed the presence of the σ_2_ and σ_4_ sigma domains in all five ECF proteins of strain B728a. The Microbial Signal Transductions (*MiST_2.1_*) database [46] was used for analysis of the ECF domain architecture and resulted in the assignment of the five B728a genes to ECF groups 18 (Psyr_0362), 11 (Psyr_0892), 5 (Psyr_1040), 7 (Psyr_1107), and 6 (Psyr_4731); the corresponding genes were subsequently designated as *ecf18*, *ecf11*, *ecf5*, *ecf7* and *ecf6* according to the nomenclature system proposed by Staroń et al. [27] (Table 2). Analysis of the ECF sigma factor protein sequences using the BLASTp program at NCBI (http://www.ncbi.nlm.nih.gov//blast/Blast.cgi) revealed the presence of homologs in other fluorescent pseudomonads with over 90% sequence homology to ECF sigma factors in other *P*. *syringae* pathovars. Table 2 summarizes the percent amino acid sequence identities between ECF sigma factors in B728a and their corresponding homologs in *P. syringae* pv. tomato strain DC3000 and *P. syringae* pv. phaseolicola strain 1448A. All five ECF sigma factors of strain B728a were highly conserved in these two pathovars of *P. syringae*, with ECF sigma factor homologies ranging from 88 to 97% amino acid identities.

**Table 2:** ECF sigma factors in the genome of *Pseudomonas syringae* pv. syringae strain B728a.

ECF sigma factor gene(ECF group^a^)	Locus tag	ECF description and associated function	Percent amino acid homology^b^
*ecf18*	Psyr_0362	RpoT-like ECF σ factor associated with toluene tolerance	94% PSPPH_0345, 94% PSPTO_5176
*ecf11*	Psyr_0892	Cytoplasmic sensing ECF σ factor	97% PSPPH_0927, 94% PSPTO_1043
*ecf5*	Psyr_1040	FecI-like ECF σ factor regulator associated with iron uptake	94% PSPPH_1093, 92% PSPTO_1209
*ecf7*	Psyr_1107	FecI-like ECF σ factor regulator associated with iron uptake	92% PSPPH_1175, 88% PSPTO_1286
*hrpL* (ECF32)	Psyr_1217	ECF σ factor regulates expression of the type III secretion genes	92% PSPPH_1294, 88% PSPTO_1404
*pvdS* (ECF9)	Psyr_1943	FecI-like ECF σ factor regulator of pyoverdine gene cluster	98% PSPPH_1909, 97% PSPTO_2133
*sigX* (ECF1)	Psyr_2096	RpoE-like ECF σ factor and likely regulator of *oprF* gene	98% PSPPH_2067, 98% PSPTO_2298
*acsS* (ECF5)	Psyr_2580	FecI-like ECF σ factor regulator of achromobactin gene cluster	98% PSPPH_2747, No PSPTO homolog
*algU* (ECF2)	Psyr_3958	RpoE-like ECF σ factor regulator of alginate gene cluster, heat-shock, and oxidative stress	100% PSPPH_3955, 99% PSPTO_4224
*ecf6*	Psyr_4731	FecI-like ECF σ factor regulator associated with iron uptake	94% PSPPH_4765, 92% PSPTO_0444

a ECF classification system according to Staroń et al. [27].

b The closest homologs found in the genomes of *P. syringae* pv. phaseolicola strain 1448A and *P. syringae* pv. tomato strain DC3000 using BLASTP at the NCBI website.

Three of the sigma factors (i.e., *ecf5*, *ecf6*, and *ecf7*) share a high degree of similarity within the σ_2_ and σ_4_ conserved domains and, accordingly, were grouped in the FecI-like sigma factor genes associated with uptake of iron complexes [27] (Table 2). Analysis of the genomic neighborhoods of these *ecf* genes placed them in operons with anti-σ factor genes encoding FecR-like transmembrane proteins (Fig. 2). All three anti-σ factor genes encode the signature anti-σ domain (ASD) at the N-terminus and a FecR domain at the C-terminus [27]. Immediately downstream of the ECF5 and ECF7 operons encoding the σ/anti-σ genes (Fig. 2) are genes encoding a FecA-like outer membrane protein that is predicted to function in uptake of extracellular iron-siderophore complexes [16]. Downstream of the ECF6 σ/anti-σ operon is an uncharacterized iron-regulated membrane protein that is not a FecA-like receptor protein. Furthermore, the *ecf6* gene of B728a is located upstream of a cluster of glycine betaine biosynthesis genes including *betA* (Fig. 2), which is predicted to encode a choline dehydrogenase [47], [48]. The genomic clustering of glycine betaine biosynthesis genes with homologs of *ecf6* was also observed in both *P. syringae* pv. tomato DC3000 and *P. syringae* pv. phaseolicola 1448A [47].

The *ecf18* gene of B728a (Table 2) is predicted to encode an RpoT-like ECF σ factor that has been associated with toluene tolerance in *P. putida* DOT-T1E [44]. Furthermore, the B728a *ecf18* gene is predicted to be located within an operon together with Psyr_0361, which encodes an anti-σ protein [47]. But unlike the ECF σ factor ortholog found in *P. putida* [44], it is not flanked by genes involved in aromatic acid biosynthesis (Fig. 2). Instead, a *cysB* homolog predicted to encode a serine O-acetyltransferase is divergently located to Psyr_0361, and a *nhaA* homolog predicted to encode a Na+/H+antiporter is located upstream of the *ecf18* gene. This chromosomal cluster of genes is conserved in the genomes of both *P. syringae* pv. tomato DC3000 and *P. syringae* pv. phaseolicola 1448A, but not in the genomes of other pseudomonad species including *P. putida* [47].

The *ecf11* gene of B728a is predicted to encode an RpoE-like sigma factor (Table 2, Fig. 2). Members of the ECF11 σ group characteristically are involved in oxidative stress responses and sensing [27], [49]. A ChrR-like anti-σ protein is predicted to be encoded by Psyr_0891 in an operon with *ecf11* [47]. A signature feature of the ChrR-like anti-σ protein is the ZAS domain located at the N-terminus of Psyr_0891; the ZAS domain is responsible for binding Zn^2+^to the anti-σ domain [27]. Flanking the operon encoding *ecf11* and the anti-σ genes are pairs of toxin-antitoxin loci (Fig. 2), which typically are associated with bacterial stress physiology [50].

### Verification of markerless deletion mutagenesis of *ecf* genes in B728a

Mutagenesis of *ecf18*, *ecf11*, *ecf5*, *ecf7*, and *ecf6* in B728a was successfully accomplished using a deletion strategy based on a modified Red recombinase deletion method [39], [51]. All five deletion mutants were confirmed by colony PCR and Southern analyses (data not shown). The Km^r^ marker was removed effectively from the resulting mutants using FLP recombination [39], [51], and the five resulting *ecf* deletion mutants are listed in Table 1. All of the *ecf* deletion mutants resembled parental strain B728a in growth rates and colony morphologies on KB agar medium, and produced the characteristic fluorescent pigment pyoverdine.

### The B728a *ecf* deletion mutants are not affected in syringomycin production

Syringomycin is a lipodepsipeptide phytotoxin produced by strain B728a that acts by forming pores in the host plasma membranes [18], [52]. Assays evaluating syringomycin production based on zones of antifungal activity to *G. candidum* revealed that the five ECF sigma factor mutants, B728aΔ*ecf5*, B728aΔ*ecf6*, B728aΔ*ecf7*, *B728aΔecf11*, and *B728aΔecf18* produced equivalent amounts of syringomycin on HMM as compared to parental strain B728a. In contrast, the syringomycin biosynthesis mutant BR132 and the global regulatory mutant B728aΔ*gacS* used as negative controls in the toxin assay [42], [53] failed to produce syringomycin. This demonstrates that the ECF sigma factor mutants are not impaired in their ability to synthesize or secrete syringomycin.

### The B728a *ecf* deletion mutants are not reduced in exopolysaccharide production, but the *ecf7* mutant exhibits a reduced swarming phenotype

B728a is known to produce at least two different exopolysaccharides, namely alginate (a co-polymer of *O*-acetylated β-1,4-linked D-mannuronic acid and L-guluronic acid) and levan (a polymer of fructofuranan) [38]. Alginate is known to promote epiphytic fitness and virulence of *P. syringae* [1], while levan acts as an extracellular storage compound that is metabolized during periods of nutrient deprivation [1], [54]. Accordingly, wild-type B728a and the five *ecf* sigma factor mutant strains were assayed on MGY medium supplemented with either sorbitol or sucrose to evaluate, respectively, differences in production levels of alginate and levan. B728a and each of the five *ecf* sigma factor mutants appeared equally mucoid on MGY containing either sorbitol or sucrose (data not shown), which was indicative of production of wild-type levels of both alginate and levan by all five of the *ecf* deletion mutants.

**Figure 3:**
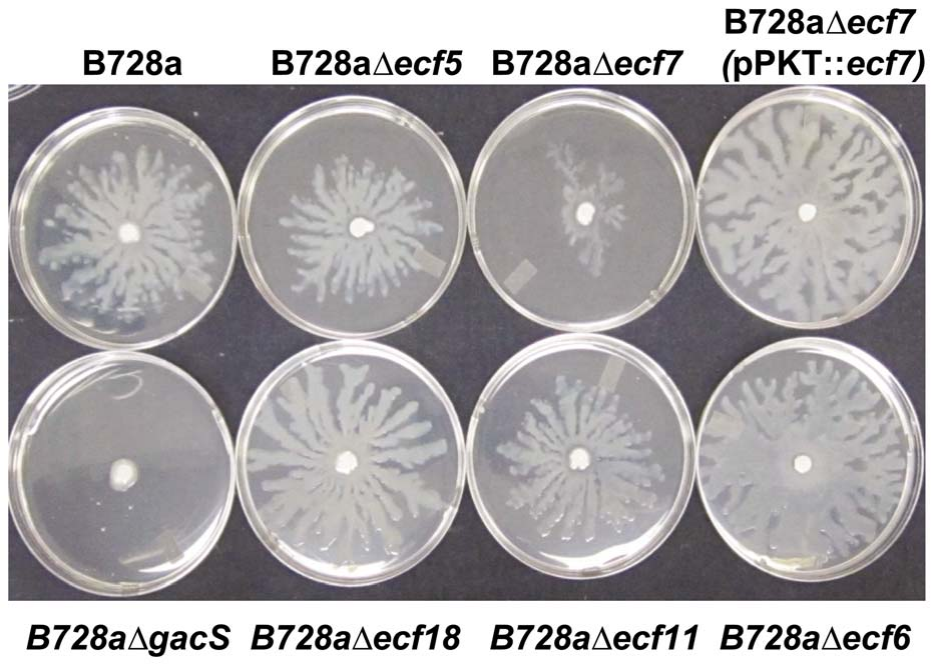
Assay for swarming activity by *P. syringae* pv. syringae ECF sigma factor mutants of strain B728a. Parental strain B728a and mutant derivatives were spotted on sterile filter discs placed in the center of semisolid NBY, and incubated in a humid chamber for 24 h at 25°C. Swarming phenotypes of B728a and the ECF sigma factor mutants, B728aΔ*ecf5*, B728aΔ*ecf7*, B728aΔ*ecf18*, B728aΔ*ecf11*, and B728aΔ*ecf6* are shown; strain B728aΔ*ecf7* carrying pPKT::*ecf7* shows restoration of the swarming phenotype. B728aΔ*gacS* is unable to swarm and was used as a negative control.

The five *ecf* deletion mutants (Table 1, Fig. 1), along with the swarming parental strain B728a and the non-swarming B728aΔ*gacS* [34], [38], were assessed for an ability to swarm on 0.4% agar medium. After inoculation onto filter disks placed at the center of a NBY semisolid agar plate, growth of B728a was observed to spread away from the disk in a dendritic pattern characteristic of swarming (Fig. 3). B728aΔ*ecf5*, B728aΔ*ecf6*, B728aΔ*ecf11*, and B728aΔ*ecf18* showed a similar movement pattern with average swarming distances ranging from 29 mm (B728aΔ*ecf5*) to 40 mm (B728aΔ*ecf6*); the average swarming distance for B728a was 35 mm. In contrast, B728Δ*ecf7* (despite growth equivalent to B728a *in vitro*) was greatly reduced in swarming activity with an average swarm distance of 18 mm (Fig. 3). Complementation of B728Δ*ecf7* with pPKT::*ecf7* fully restored the ability to swarm to a distance of 40 mm. No swarming movement occurred for B728aΔ*gacS* on the semisolid NBY agar medium.

### The B728a ECF sigma factor mutants are fully virulent causing typical brown spot disease symptoms on bean, reaching high levels of multiplication *in planta*

The influence of ECF sigma factors on development of bacterial brown spot disease in bean plants was measured by standard pathogenicity assays [17], [38] of the five *ecf* deletion mutants at an initial inoculum concentration of 10^6^ CFU/ml. Visual observations of foliar symptoms at 2-day intervals showed that the five *ecf* mutants were equivalent to B728a in ability to cause typical brown spot disease lesions. Small water-soaked lesions were visible at day 2; lesions by day 6 had coalesced to cause widespread necrosis of the infected bean leaf. For example, bean leaves inoculated with B728aΔ*ecf5* and B728a*Δecf7* mutants are shown (Fig. 3A) 4 days after inoculation as compared to B728a. In all cases, the B728aΔ*gacS* mutant failed to cause brown spot disease lesion on bean and served as an internal control for the pathogenicity assays [38].

**Figure 4:**
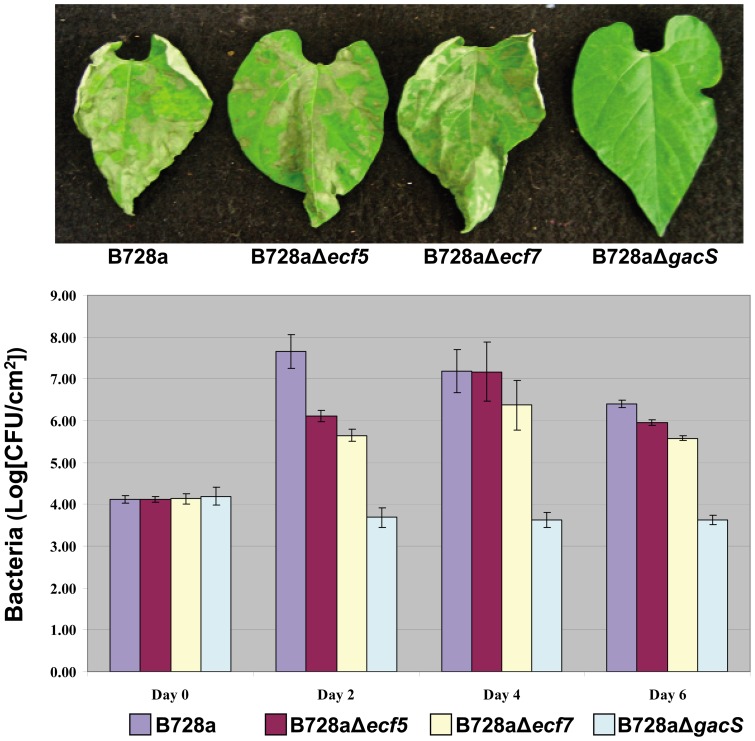
Pathogenicity assays to evaluate the contribution of the *ecf5* and *ecf7* genes to disease development in a susceptible bean (*Phaseolus vulgaris* cv. Blue Lake 274) host. (A) Disease symptoms. Bean leaves were inoculated using vacuum infiltration with cell suspensions containing 10^6^ CFU/ml of strain B728a, ECF sigma factor mutants B728aΔ*ecf5* and B728aΔ*ecf7*, and the avirulent B728aΔ*gacS* as a negative control. Plants were maintained at 25°C in a growth chamber for 6 days. The experiment was performed twice; representative results are shown 4 days after inoculation. **(B) Bacterial populations.** Bean leaf populations of the four bacterial strains identified in panel A were determined at days 0, 2, 4 and 6 days after inoculation with 10^6^ CFU/ml for each strain. Bacterial populations are shown in terms of the logarithm of CFU/cm^2^ of leaf surface. Values are the average counts from four individual plants sampled at each time point; the experiment was repeated on two occasions. Error bars represent the standard errors (SE) of the respective means.

Bacterial populations in infected plants were monitored over a 6-day period to discern possible differences in apoplastic growth of *ecf* deletion mutants in bean leaves. Results show that the populations of all five *ecf* mutants reached similar levels of approximately 10^7^ CFU/cm^2^ at 4 days post-inoculation with the population remaining stable through day 6. Typical results for leaf colonization of two *ecf* deletion mutants, B728aΔ*ecf5* and B728a*Δecf7*, are shown in Fig. 4B. At 2 days post-inoculation, bacterial titers for B728a were 5×10^7^ CFU/cm^2^, whereas populations for B728aΔ*ecf5* and B728aΔ*ecf7* indicated a slight delay in population growth based on average recoveries of 1.3×10^6^ CFU/cm^2^ and 4.5×10^5^ CFU/cm^2^, respectively. In contrast, the population of B728aΔ*gacS* was measured at 5×10^3^ CFU/cm^2^ at day 0 and remained effectively unchanged throughout the 6-day period. At 4 days post-inoculation, populations of B728aΔ*ecf5* and B728aΔ*ecf7* were approximately 10^7^ CFU/cm^2^, which is not significantly different from the populations reached by B728a. These results indicate that the ability of B728aΔ*ecf5*, B728aΔ*ecf7*, and other *ecf* deletion mutants to grow *in planta* may be delayed as measured by populations at day 2 (Fig. 3B), but ultimately population growth of these mutants was indistinguishable to that of parental strain B728a at 4 days post-inoculation.

### Sensitivity of a B728aΔ*ecf11* mutant to oxidative stress and a B728aΔ*ecf18* mutant to toluene

Comparisons of the sensitivity of B728a to B728aΔ*ecf11* to oxidative stress by the disk diffusion method showed no significant difference in sensitivity to H_2_O_2_. Measurements of zones of inhibition averaged 19.0 ±0.7 mm for B728a and 20.7 ±0.6 mm for B728aΔ*ecf11*. Likewise, the B728aΔ*ecf18* mutant was indistinguishable from B728a for toluene sensitivity as measured by growth and survival in the presence of 0.3% (vol/vol) toluene (data not shown).

### The *ecf5*, *ecf6*, and *ecf7* genes encoding FecI-like sigma factors control expression of genes involved in iron transport in B728a

The genomic context for the *ecf5, ecf6,* and *ecf7* shows the occurrence of specific siderophore receptors and transmembrane sensors adjacent to these ECF sigma factor genes (Fig. 2). For example, the *ecf7* locus shows the presence of a heme oxygenase gene (Psyr_1104, *hemO*) predicted to be involved in the uptake of heme, which acts as an essential source of iron for pathogenic bacteria such as *P. aeruginosa* [13]. After uptake, the heme molecule is degraded by heme oxygenases to yield ferrous ion for utilization by the cell [55]. The *ecf7* gene showed a significant level of expression in low iron conditions by qRT-PCR analysis (Table 3) along with the linked heme oxygenase gene *hemO*, a *fecA*-like outer membrane receptor (Psyr_1105), and a FecR-like anti-σ gene (Psyr_1106). The *hemO* gene showed the highest level of transcript abundance under low iron conditions, which was over 150 times higher under low iron conditions as compared to expression under high iron levels (Table 3). Furthermore, qRT-PCR analysis indicated a significant increase in expression of *ecf5* and *ecf6* along with the associated Psyr_1038 and Psyr_4729 genes, and the FecR-like anti-σ genes, Psyr_1039 and Psyr_4730, in a low iron medium (Table 3). All of these genes were suppressed under conditions of high iron concentration (Table 3). This demonstrates a significant role of iron in expression of the three FecI-like *ecf* σ factor genes and supports an associated function in iron uptake and transport.

**Table 3:** Expression analysis in strain B728a of three FecI-like ECF σ factor genes (*ecf5*, *ecf7*, and *ecf6*) and associated putative iron-responsive genes in low and high iron media.^a^

Gene	Fold change in transcript levels±SE	
	Expression in low iron	Expression in high iron
*ecf5* (Psyr_1040)	10.87±1.83	−4.39±0.12
Psyr_1038	5.61±0.22	1.08±0.17
Psyr_1039	14.54±0.18	2.36±0.07
*ecf7* (Psyr_1107)	8.39±0.56	1.84±0.28
Psyr_1104	426.80±0.14	2.76±0.36
Psyr_1105	154.47±0.40	3.28±0.61
Psyr_1106	4.21±0.51	2.15±0.11
*ecf6* (Psyr_4731)	31.61±0.12	1.11±0.41
Psyr_4729	22.90±0.41	1.27±0.08
Psyr_4730	20.24±0.22	−1.25±0.07

a Values represent the average fold differences of three technical replicates of three biological samples. Gene expression was normalized to the *16s-rRNA* and *recA* internal control genes. Fold changes for expression in iron-limited HMM were compared to cells grown in HMM supplemented with 10 µM iron. Fold changes for expression in HMM supplemented with 100 µM iron were compared to cells grown in HMM supplemented with 10 µM iron. Negative values indicate a decrease in expression levels as computed by taking the negative inverse of a fold change value less than 1.

Because FecR-type genes are predicted to encode anti-sigma genes and are located within an operon with their cognate sigma factor gene in B728a (Fig. 2), qRT-PCR expression analysis of Psyr_1039, Psyr_4730, and Psyr_1106 showed that they essentially were not expressed in the corresponding ECF sigma factor deletion mutant (Table 4). These FecR-type genes were also down-regulated or not expressed in several of the other sigma factor mutants. Greater reductions in expression were observed for the FecA-like gene Psyr_1105 and the Psyr_4729 gene, encoding a PepSY-associate™helix protein, in their respective *ecf* deletion mutant. The influence of the B728aΔ*ecf7* mutation on expression levels was most apparent for its accompanying FecA-like gene Psyr_1105 and the *hemO* gene (Psyr_1104) with more than a 160-fold decrease in expression relative to strain B728a. Transcript levels of the FecR- and FecA-like iron-responsive genes were essentially unchanged in a B728aΔ*gacS* mutant as compared to B728a, demonstrating that the GacS global regulator did not influence expression of these genes associated with ECF sigma factors. Nevertheless, *hemO* expression was about 3-fold lower in B728Δ*gacS* in comparison to B728a. Taken together, these data suggest a functional overlap in the regulons of Ecf5, Ecf7, and Ecf6.

**Table 4:** Expression analysis in low iron media of putative iron-responsive genes in B728aΔ*ecf5*, B728aΔ*ecf6*, and B728aΔ*ecf7* mutant strains as compared to B728a.^a^

Gene	Fold change in transcript levels±SE			
	B728aΔ*ecf5*	B728aΔ*ecf7*	B728aΔ*ecf6*	B728aΔ*gacS*
Psyr_1038	−3.61±0.23	−3.57±0.03	−1.85±0.42	1.39±0.50
Psyr_1039	−3.41±0.15	−5.47±0.49	−4.25±0.19	−1.51±0.09
Psyr_1104	−6.73±0.14	−220.27±1.40	−7.92±0.44	−2.98±0.14
Psyr_1105	−2.57±0.24	−166.11±2.62	1.48±0.24	−1.09±0.30
Psyr_1106	−2.50±0.02	−1.50±0.03	−2.12±0.14	1.87±0.26
Psyr_4729	−2.41±0.26	1.44±0.06	−27.64±0.30	−1.54±0.54
Psyr_4730	−2.81±0.14	−1.11±0.07	−4.06±0.34	−1.24±0.05

a Values represent the average fold differences of three technical replicates of three biological samples. Gene expression was normalized to the *16s-rRNA* and *recA* internal control genes. Negative values indicate a decrease in expression levels as computed by taking the negative inverse of a fold change value less than 1.

### The B728a *ecf7* regulon includes genes associated with Cup fimbriae assembly in B728a

**Figure 5:**
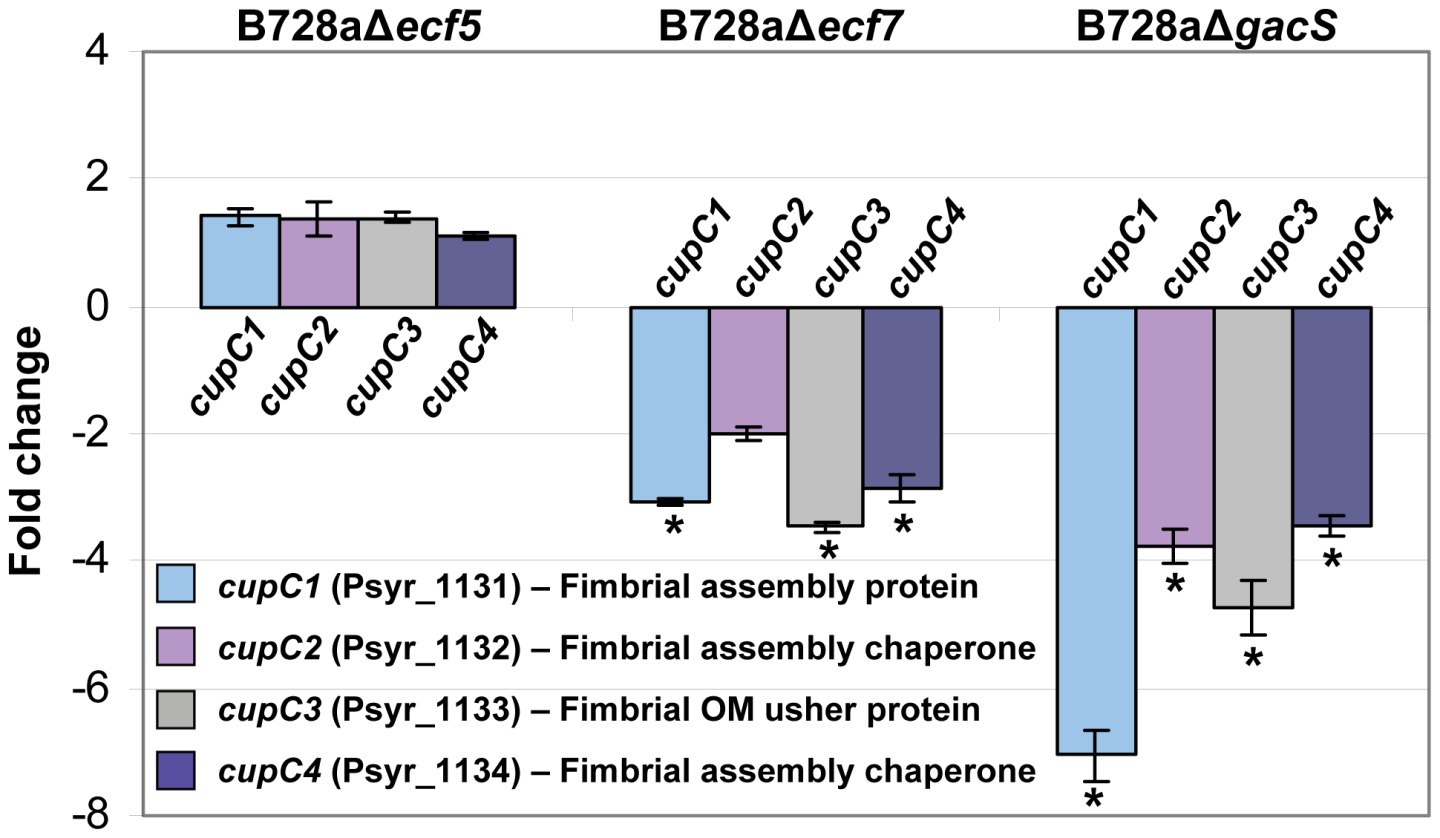
Quantitative real-time PCR analysis of Type I fimbrial gene expression in Δ*ecf5* and Δ*ecf7* mutants of B728a. The pilus assembly/fimbrial biogenesis gene cluster (Psyr_1131-Psyr_1134) analyzed is located in close proximity to the *ecf7* sigma factor gene. Values represent the average fold differences in gene expression from parental strain B728a; results are averages of three technical replicates (to measure reproducibility from a single source) from each of three biological samples grown in NBY liquid medium at 25°C (∼5×10^8^ CFU/ml final concentrations). Gene expression levels were normalized to the *16S-rRNA* and *recA* internal control genes, and standard deviations from the mean are denoted by the error bars; asterisks denote greater than a 2-fold change in gene expression. A Student’s *t*-test was performed using 95% confidence interval to calculate *p*-values between biological replicates. Negative values indicate a decrease in expression levels as computed by taking the negative inverse of a fold change value less than 1.

Investigation of the genomic regions flanking *ecf7* revealed the occurrence of a cluster of five genes (Psyr_1131–1135) involved in fimbriae assembly and organized in two operons (Fig. 5). The first operon carries the fimbrial assembly protein and chaperone genes (*cupC1* and *cupC2*), and the second operon carries the fimbrial outer membrane usher protein, a fimbrial assembly protein (*cupC3* and *cupC4*), and a third protein of unknown function. These genes are orthologous to genes described for *P. aeruginosa* that encode a chaperone-usher pathway (Cup) demonstrated to form fimbrial structures important in promoting biofilm formation and cell clustering [56]. Comparative sequence analysis of these fimbriae-associated proteins also revealed the presence of orthologs of the *cupC* genes in *P. syringae* pv. tomato DC3000 and *P. syringae* pv. phaseolicola 1448A (Table 5). Because pili have been implicated in swarming behavior of *P. aeruginosa* [57], and because Ecf7 influences swarming ability in B728a (Fig. 3), we determined the role of *ecf7* on the expression of the pilus-associated genes. The transcript abundance was measured by qRT-PCR analysis of Psyr_1131–1134 in B728a and mutant derivative strains. B728aΔ*ecf7* exhibited a significant decrease in transcript levels of all the *cupC* genes with a 2-fold or more reduction in expression of the fimbrial gene cluster. In contrast, B728aΔ*ecf5* did not show any apparent change in transcript levels of these fimbriae-associated genes (Fig. 5). Interestingly, the B728aΔ*gacS* mutant likewise showed a reduction in expression levels of the fimbrial genes with a 7-fold decrease in expression of the *cupC1* gene (Psyr_1131) predicted to encode a fimbrial subunit protein [56]. These results indicate that *ecf7* positively regulates expression of Psyr_1131–1134 predicted to encode Cup fimbriae.

**Table 5:** Putative pilus assembly/fimbrial genes downstream of *ecf7* in B728a.^a^

*P. syringae* pv. syringae B728a	*P. syringae* pv. phaseolicola 1448A	*P. syringae* pv. tomato DC3000	*P. fluorescens* Pf-5
Psyr_1131 (CupC1, fimbrial protein)	PSPPH_1199 (95%)	PSPTO_1312 (94%)	PFL3922 (31%)
Psyr_1132 (CupC2, fimbrial assembly chaperone)	PSPPH_1200 (83%)	PSPTO_1313 (73%)	PFL3923 (43%)
Psyr_1133 (CupC3, fimbrial usher protein)	PSPPH_1201 (85%)	−	PFL3924 (49%)
Psyr_1134 (CupC4, fimbrial assembly chaperone)	−	PSPTO_1317 (68%)	−

a Percent amino acid identities of orthologs are shown in parentheses.

## Discussion

The discovery that ECF sigma factors frequently mediate responses to environmental signals and sometimes control genes of critical importance to virulence, such as HrpL in controlling the T3SS of *P. syringae* [19], [58], has led to speculation that other ECF sigma factors encoded by the *P. syringae* genome may likewise control gene targets critical to survival in the plant environment [10]. Furthermore, ECF sigma factors account for two-thirds of the sigma factors encoded by the genome of *P. syringae* pv. syringae strain B728a (Fig. 1). Of the 10 ECF sigma factors of B728a, five belong to the FecI-type and are predicted to be involved in the regulation of iron transport systems [10]. This includes two ECF sigma factors vital to siderophore production by strain B728a, namely PvdS [17], [26] and AcsS [17], respectively involved in the regulation of pyoverdine and achromobactin production. In this study, we characterized the remaining three FecI-like ECF sigma factor genes (*ecf5*, *ecf6*, and *ecf7*) along with two others (*ecf18* and *ecf11*) that are not responsive to environmental iron levels. All five proteins display domains 2 and 4, which are hallmark signatures of ECF sigma factors [5], [6], and were classified into distinct ECF sigma factor protein families as described by Staroń et al. [27]. The classification system was a convenient basis for further characterization of the ECF sigma factor genes relative to their genomic context.

Examination of five *ecf* deletion mutants for measurable changes in virulence as observed by symptom development and apoplastic growth within bean leaves revealed that none of the sigma factor genes are required for *in planta* growth and lesion formation. The only measurable effect was a delay of population growth at day 2 for two of the mutants, B728aΔ*ecf5* and B728aΔ*ecf7*; they eventually recovered to a final population of ∼10^7^ CFU/cm^2^ by day 4 that was equivalent to the population of parental strain B728a. Consequently, of the 10 ECF sigma factors of B728a, it appears that only HrpL, which activates the T3SS, has a major regulatory role in controlling virulence in *P. syringae* [15], [19]. AlgU activates genes dedicated to alginate production in *P. syringae* and contributes to *in planta* growth and survival of *P. syringae* pv. glycinea [59], and presumably has a similar function in other pathovars [20]. In contrast, the two siderophore-associated sigma factor genes, *pvdS* and *acsS*, show little or no effect [17], [26], [60] on lesion formation or apoplastic growth in susceptible plant hosts. Correspondingly, Jones and Wildermuth [61] demonstrated that *P. syringae* pv. tomato DC3000 disrupted in ability to produce high affinity siderophores was fully pathogenic in the leaf apoplast, which indicated that ample iron is available to support *in planta* bacterial growth of *P. syringae*.

An earlier study by Wang et al. [30] demonstrated that disruption of *rpoS*, a gene which frequently controls the transcription of secondary metabolite genes in stationary phase bacterial growth [3], did not affect the production of either syringomycin or syringopeptin. The predicted −10/−35 promoter region of the *syrB1* biosynthesis gene was established by deletion and site-directed mutagenesis analysis, and the presence of σ^70^-like promoter sequence was seen for all *syr-syp* genes and operons [30]. Furthermore, a conserved *syr-syp* box with dyad symmetry around the −35 region was identified by computer analysis of *syr-syp* genes and operons. Consequently, a goal of our sigma factor gene analyses was to determine if any one of the five *ecf* genes regulated the *syr-syp* genes and operons dedicated to phytotoxin production. The results demonstrated that none of the five *ecf* genes influence syringomycin production. Correspondingly, earlier evidence indicated that the ECF sigma factor genes *hrpL*, *pvdS*, *algU*, *sigX*, and *acsS* did not regulate toxigenesis in *P. syringae* [17], [43]. We surmise that the major σ^70^ encoded by the *rpoD* gene is the sigma factor responsible for the transcription of *syr-syp* genes in *P. syringae*. This is supported by the report of Schnider et al. [62] that antibiotic production by *P. fluorescens* is controlled by the RpoD sigma factor.

Expression analyses of the *ecf5*, *ecf6*, and *ecf7* genes in low and high iron media indicate that these FecI-type ECF sigma factors are indeed influenced by iron stress in the extracellular environment (Table 3), and are co-expressed with FecR-like genes encoding cognate anti-sigma factors. The function of the anti-sigma protein is to tightly bind the cognate ECF sigma factor in the absence of a stimulus and maintain σ inactivity [27]. Moreover, qRT-PCR expression profiles of the *ecf5* and *ecf7* associated FecA-like outer membrane receptors (Psyr_1038 and Psyr_1105), the *ecf6* iron-regulated membrane protein Psyr_4729, and the FecR-type sensors (Psyr_1039, Psyr_4730, and Psyr_1106, respectively) were up-regulated in iron-deficient conditions and down-regulated in iron-replete conditions (Table 3). The *hemO* gene (Psyr_1104; Fig. 2), which is predicted to encode a heme oxygenase, showed the highest level of transcript abundance under low iron conditions (Table 3), and was demonstrated to be regulated by the *ecf7* gene (Table 4). Recently, Lim et al. [63] reported that *P. fluorescens* Pf-5 has both a sigma factor (PFL_4625) gene and a *hemO* (PFL_4628) gene that were transcriptionally upregulated under iron-limited conditions; these genes are orthologous to the *ecf7* and *hemO* genes of B728a. The glycine betaine biosynthesis gene *betA* located upstream of *ecf6* was unchanged in expression in B728aΔ*ecf6* as compared to B728a (data not shown). Firoved et al. [64] determined that the glycine betaine biosynthesis genes are controlled by AlgU in *P. aeruginosa*, and we assume that AlgU similarly controls expression of glycine betaine genes in *P. syringae* B728a. Other than controlling expression of the cognate anti-sigma and FecA-like proteins, the regulatory targets of *ecf6* remain unknown.

A recent study by Markel et al. [65] used ChlP-seq to investigate the regulons in *P. syringae* pv. tomato DC3000 associated with ECF sigma factors encoded by PSPTO_0444, PSPTO_1209, and PSPTO_1286, which are orthologous to the *ecf6*, *ecf5* and *ecf7* genes of B728a, respectively. In addition to PSPTO_0446 encoding an uncharacterized iron-regulated membrane protein (orthologous to Psyr_4729 in B728a), they discovered that the PSPTO_0444 sigma factor in DC3000 bound a FecA-like receptor gene (PSPTO_4128) located at a distance over 2.2 MB from the sigma factor gene. The regulon controlled by PSPTO_1286 included a heme oxygenease (PSPTO_1283) and a closely linked FecA-like receptor (PSPTO_1284). Likewise, we observed that the ECF7 sigma factor, which is orthologous to PSPTO_1286, regulated a heme oxygenase (Psyr_1104) and FecA-receptor (Psyr_1105) in B728a (Table 4). The regulon associated with PSPTO_1209 was somewhat larger and included the type VI protein secretion system, an OmpA-family protein, and a helicase family protein in addition to a FecA-like receptor [65]. Ferric uptake regulator binding sites (Fur box) were identified upstream of FecA-like receptor genes [65], [66], which shows the importance of iron regulation on expression of these sigma factors and their downstream regulon targets. Consequently, it is likely that Fur controls expression of the FecI-like sigma factors and their associated regulons in B728a as has been previously demonstrated in DC3000 [65], [66].

Assays conducted with the ECF sigma factor mutants revealed that B728aΔ*ecf7* was reduced by ∼50% in its ability to swarm on semisolid agar surfaces (Fig. 3), a phenotype which could be fully complemented by the *ecf7* gene *in trans*. Furthermore, orthologs of genes encoding a functional chaperone-usher pathway (Cup) fimbrial structure are located in close proximity to *ecf7* and were demonstrated to be down-regulated in the B728aΔ*ecf7* mutant (Fig. 5). The chaperone-usher pathway has been described for the assembly of fimbrial adhesins in *P. aeruginosa* that help facilitate bacterial attachment to the host and promote biofilm formation [56]. We also observed that the two-component global regulator gene, *gacS*, controlled expression of the *cupC* genes; the *cupC3* gene expression was down four-fold in B728aΔ*gacS*. This observation is consistent with the report by Burrowes et al. [67], who conducted transcriptome profiling of an *rsmA* mutant of *P. aeruginosa* PAO1 and observed that expression of the *cupC3* usher gene was down three-fold compared to PAO1. It is well established that the GacS/GacA two-component system targets the levels of RsmA protein which in turn controls expression of genes critical to biofilm formation [68]. We speculate that GacS/GacA/RsmA signal transduction system, which controls expression of the *pvdS* sigma factor gene [67], also regulates expression of the *ecf7* sigma factor gene to influence expression of the *cupC* genes in B728a. Consequently, it appears that the expression of the Cup fimbrial biogenesis genes in B728a contributes to the complex phenomenon of swarming in *P. syringae* B728a, and may play a vital role in the mobilization of bacteria to different environments on the leaf and biofilm formation [57].

Two of the B728a ECF sigma factors characterized were not regulated by iron and were named *ecf11* and *ecf18* according to the nomenclature system described by Staroń et al. [27]. ECF18 is orthologous to the RpoT ECF factor originally described for *P. putida* and involved in toluene tolerance (annotated as PP3006) [44]. The RpoT regulon of *P. putida* controls a limited number of transcriptional units including the TtgGHI efflux pump responsible for resistance to toluene and other organic solvents. Comparison of the toluene tolerance of B728aΔ*ecf18* to B728a, however, did not demonstrate differences in toluene sensitivities (data not shown). Ecf11 is orthologous to the *rpoE* gene of *P. syringae* pv. phaseolicola 1448A [47] and has been associated with oxygen stress in *Rhodobacter sphaeroides* [49]. As with all group ECF11 sigma factors, the *ecf11* gene of B728a is linked in an operon to a ChrR-like anti-sigma gene, Psyr_0891 [47]. A characteristic cupin-like fold, which is the likely site of redox sensing [27], is observed in the C-terminal domain of the anti-sigma factor protein. Nevertheless, assays of B728aΔ*ecf11* for increased sensitivity to oxidative stress relative to B728a did not show measurable differences.

Specialized ECF factors are associated with bacterial responses to various environmental stimuli and stresses [2], [3]. The occurrence of 10 ECF sigma factors in the genome of *P. syringae* pv. syringae B728a suggests vital regulatory roles for successful establishment of the bacterium in the plant host environment. This is well documented for HrpL and AlgU, which contribute to plant pathogenicity by respectively activating T3SS genes [19], [58] and the alginate biosynthesis genes [20]. In addition, PvdS and AcsS activate genes associated with production of the pyoverdine [26] and achromobactin [17] siderophores, respectively. Our analysis of deletion mutants of five previously uncharacterized ECF sigma factor genes in B728a showed surprisingly little to no effect in virulence and other phenotypic assays. Most notably, B728aΔ*ecf7* exhibited a reduced ability to swarm and was downregulated in expression of CupC fimbrial genes. Transcriptomic analysis, such as RNA-seq [17], of the B728a Δ*ecf* mutants should provide additional leads as to the regulatory networks or stimulons controlled by the ECF sigma factors.

## Supporting Information

Table S1 **Primers used for PCR amplification.**
(DOCX)

Table S2 **Primers used for qRT-PCR analysis.**
(DOCX)
