# Supplementary material for: Characterization of Five ECF Sigma Factors in the Genome of Pseudomonas syringae pv. syringae B728a
Source: PLoS One. 2013 Mar 14;8(3):e58846. doi: 10.1371/journal.pone.0058846 (PMC3597554; doi:10.1371/journal.pone.0058846)
Supplement: Table S1 — Primers used for PCR amplification. (DOCX) [file pone.0058846.s001.docx]

**Table S1.** Primers used for PCR amplification

| **Name** | **Sequence (5’ – 3’)** | **Source** |  |
| --- | --- | --- | --- |
| Prr1040F | CACCCCAATTGCGTACGCCCATTCAGTT | This study |  |
| Prr1040R | GCCCTGCGATTTGATTCCATGTGT | This study |  |
| Prr1107F | CACCCGTCATCACTGACCTTGTAGTAGC | This study |  |
| Prr1107R | AACTGACCTGATACAGCAACAGC | This study |  |
| 1107compF  1107compR  Prr1040KmF | ATATCAGAATTCTGATCCATTGCAGC  ATATCAGAATTCTCCAGACAAGCGCC  GGATTTGTTTAAAGACTCGTTGTTCGGATTCATAGGGTGTAGGCTGGAGCTGCTTCG | This study  This study  This study |  |
| Prr1040KmR | GAATCCACGCCACCAATGAGTCCCCGTGGATTGTTTATTCCGGGGATCCGTCGACC | This study |  |
| Prr1107KmF | CGATATGAGTGTGGCAGCGTGCGGCAGGGTTCAGGGGTGTAGGCTGGAGCTGCTTCG | This study |  |
| Prr1107KmR | GCGGACAAATATTTACAGATCCAGGCTTGACCCTCCATTCCGGGGATCCGTCGACC | This study |  |
| Prr1041F | AAACATGCGCGCGAAGTGGTATTC | This study |  |
| Prr1041R | AAAGCGAAAGGTTCATGGTGCAGG | This study |  |
| Prr1108F | TCTGTCCTTTACTGCGCGTTATGC | This study |  |
| Prr1108R  Prr0362F  Prr0362R  Prr0892F  Prr0892R  Prr4731F  Prr4731R  Prr0362KmF  Prr0362KmR  Prr0892KmF  Prr0892KmR  Prr4731KmF  Prr4731KmR  0362F  0362R  0892F  0892R  4731F  4731R | ACCGTCAATGGTGACCGTATTTCG  CACCACGAGAACGACATTCCAGTGCAGA  GAAGCGGGCAAGCTGATGTTTGAT  CCCTACCGGTTATCGCGCTACGGATTT  AGGCTTTTGAAGCATGTCCACGAAC  CACCCGTTCAATCTTCTGCTGCTG  TACCGAACGGCGTATTCAAC  AGCCAGCTCGTGAATGTTGTCGTCTAAAGGCCGGGGTGTAGGCTGGAGCTGCTTCG  AACGAGGCAGTAGTCTTCCATTGATGGCGGATCATCATTCCGGGGATCCGTCGACC  TAACGAGATGACCTGCGACAGCGCTCCTGCGGCATAGTGTAGGCTGGAGCTGCTTCG  TGCATCGCGAACCAAACCCACACAATGGTGATCAGAATTCCGGGGATCCGTCGACC  TTGAGCACACGGACAGAGACCGGTCTGGTGTTCACGGTGTAGGCTGGAGCTGCTTCG  AGATCCTCCTCCCGACAGACGCGAATTGCCACTACCATTCCGGGGATCCGTCGACC  GTACATACTCGCCAGCCAGCTCGTGAATGTTGTCGT  AACGAGGCAGTAGTCTTCCATTGATGGCGGATCATC  TAACGAGATGACCTGCGACAGCGCTCCTGCGGCATA  TGCATCGCGAACCAAACCCACACAATGGTGATCAGA  TTGAGCACACGGACAGAGACCGGTCTGGTGTTCACG  AGATCCTCCTCCCGACAGACGCGAATTGCCACTACC | This study  This study  This study  This study  This study  This study  This study  This study  This study  This study  This study  This study  This study  This study  This study  This study  This study  This study  This study |  |
