# Supplementary material for: Characterization of Five ECF Sigma Factors in the Genome of Pseudomonas syringae pv. syringae B728a
Source: PLoS One. 2013 Mar 14;8(3):e58846. doi: 10.1371/journal.pone.0058846 (PMC3597554; doi:10.1371/journal.pone.0058846)
Supplement: Table S2 — Primers used for qRT-PCR analysis. (DOCX) [file pone.0058846.s002.docx]

**Table S2.** Primers used for qRT-PCR analysis

| **Name** | **Sequence (5’ – 3’)** | **Source** |  |
| --- | --- | --- | --- |

| qRT1040F | TAGTGATTGCACAGCACACG | This study |
| --- | --- | --- |
| qRT1040R | CACACTGTCGAAGGCCTGTA | This study |
| qRT1107F | GTCTGGAAAACGAAGGGTTC | This study |
| qRT1107R | GTCGCACTCAGACTTCAACA | This study |
| qRT1038F | GTGTAGTACTGGCGGTTGAAGATG | This study |
| qRT1038R | CTTAATTTCGACAGCGCCTTCCAG | This study |
| qRT1039F | AGCGACTCAACTCTGCGAGAAA | This study |
| qRT1039R | ATTTGCCGGAGCCCTTGAAATAGC | This study |
| qRT1104F | GTTTGATCAGGAATGCTGCACCGA | This study |
| qRT1104R | TCAAACGATCATCAGTGACCTGCC | This study |
| qRT1105F | GAAGGTCCAGTCATTCCAGCTGAT | This study |
| qRT1105R | CAACGATCAAGCGCCAGAAAGTGA | This study |
| qRT1106F | ATCGAAGAAGGCTTCACCCTGGTA | This study |
| qRT1106R | GTTGTTCAGCTTCAGCAATCTGCC | This study |
| qRT1131F | ACAACGGCACAATGGTCTTCAACG | This study |
| qRT1131R | TCGAATCGGCTTTCGCTCGATACA | This study |
| qRT1132F | TGACACTGAACTTGCCCTTCTGGA | This study |
| qRT1132R | TAACCGGTTGTTTGTTGCCAAGGG | This study |
| qRT1133F | AGACGTTCACCGATTCGCAGGTAT | This study |
| qRT1133R | AACTTCCACGGTCGCATTGGTTTC | This study |
| qRT1134F | CACCTGTATGTGCTTGAGATACCC | This study |
| qRT1134R | TCATGAGTCAGTGTCCACAGCA | This study |
| qRT0362F  qRT0362R  qRT0892F  qRT0892R  qRT4731F | TCATCATGAACAGGCGACACTGGA  CTGCGCGATTTCCTGATGCGAATA  GTTGTTTCGCATCGCACGCAATCT  TGGATGGCGATGTTGAGTTGTTGC  ATGAATTGCTTGCGCCCAGAGAAC | This study  This study  This study  This study  This study |
| qRT4731R | TGGCCTTGAGGTCTTCCACAATCA | This study |
| qRT0889F  qRT0889R  qRT0890F  qRT0890R  qRT0894F  qRT0894R  qRT0895F  qRT0895R  qRT4729F  qRT4729R  qRT4730F | CGCTCAAACCCGCTAAAGCAAAGT  CATTCGGCCAATTCTGTGGGTTCA  TTGATTCTGACGCCTGCGGCTTAT  ATCTGGTTGCAAAGAACGACACCC  TAAAGAACAAGCCAGCTTCGGTGC  AACAACGGCAAGATTCCTTTCGGG  TCGATGTTATAGCCGTGGGCAGAA  GCGCTCTGCCATGAAATCATCACT  TTTGCACCCTGTTCCTGTTGATGC  TCGGCAGGCATCTCTTTGTAATGC  TCTGCAGGACGGTGAAATACTGGT | This study  This study  This study  This study  This study  This study  This study  This study  This study  This study  This study |
| qRT4730R  qRT4732F  qRT4732R  qRT4733F  qRT4733R  qRT4734F  qRT4734R  qRTrecAF  qRTrecAR  qRT16SF  qRT16SR | CAGAACCTGTTCACCGGAAGCATT  AAGCTGGATATCCCGGTGGTTCAT  ACTTCAGCGAAGGATACAGCGACA  TGAAAGCCGAGTTCGAAGCGAAGA  TTTCCATGTGCTCGAAGCTGACCA  CCTGCAAGTGATCATCGAAGGCAA  ACAAACGGTGGTCGTTGATACGCT  CTTCGGTACGCCTGGACA  ACACCGCCCGTCACACCA  ATCATCATGGCTGGCTGGAAAGC  TCAAGGCTGCGACGAGTGTAGAAA | This study  This study  This study  This study  This study  This study  This study  [[17](#_ENREF_17),[38](#_ENREF_38)]  [[17](#_ENREF_17),[38](#_ENREF_38)]  [[17](#_ENREF_17),[38](#_ENREF_38)]  [[17](#_ENREF_17),[38](#_ENREF_38)] |

______________________________________________________________________________
